# Psychotherapy, formulation and welfare: Themes of the 2023 trainee article collection update

**DOI:** 10.1177/10398562241231863

**Published:** 2024-02-07

**Authors:** Michael Weightman

**Affiliations:** Adelaide, SA

Dear Editor,

The *Australasian Psychiatry* trainee article collection continues to evolve as new content enters the journal. This collection brings together all past content from the journal directly relevant to those undertaking psychiatry training in Australia and New Zealand: the aims and methodology behind this resource have been described previously^1^ and the complete list of articles is hosted on the journal’s website (https://journals.sagepub.com/page/apy/virtualspecialcollections).

Within Volume 31, an additional 11 articles and two letters were identified as suitable for inclusion into the online collection. Table 1 details the titles of the included papers, with the full citations available online. Only content relevant to current trainees was included, which necessitated exclusion of papers addressing the removal of the OSCE or discussing the development of future advanced training certificates.

**Table 1.** Snapshot of additional resources from Volume 31 of *Australasian Psychiatry*

Category	Article title
General training	• Is workplace-based assessment achievable in practice?^a^
Scholarly project	• The importance of appraising articles and conducting psychiatric journal clubs
Psychotherapy written case	• ‘To see or not to see, that is the question’ – A commentary on patient selection for the RANZCP psychotherapy written case
	• A guide to support trainee’s success in the psychotherapy written case submission
	• Psychodynamic practice in psychiatry
Formulation	• Considering patient narrative-based and medico-scientific epistemologies in framing psychiatric care
	• Formulation in psychiatry – a guide to developing competence
	• Addressing the dauntingness of formulation: The value of considering empathy as “grasping”
Supervision	• From the chemical imbalance to the power imbalance: A psychiatry trainee’s perspectives on service-user supervision
Trainee welfare	• Psychiatrist and trainee moral injury during the organisational long COVID of Australian acute psychiatric inpatient services
	• The ethics of sexual relationships between psychiatrists and trainee psychiatrists in the ‘me too’ era
	• Advancing psychiatry trainee wellbeing and safety: Building on RANZCP Position Statement 48

^a^This entry includes both the original letter and a subsequent reply letter outlining the RANZCP Education Committee’s response.

The included articles from 2023 covered a diverse range of categories (Table 1). In particular, the psychotherapy written case was the subject of several articles, including important instructional papers addressing both the crucial step of patient selection and guidance on approaching the final written case report. These articles form a neat pairing covering both the beginning and conclusion of what is a significant, and often challenging, assessment task for trainees.

There were also articles addressing skills relevant to formulation, which is frequently identified as a particularly daunting and difficult task for trainees.^2^ The three new articles from this year complement the extensive list of articles on formulation already in the collection. As with recent years, there are new articles covering aspects of trainee welfare; such as moral injury, ethical boundaries, and the College’s position statement on wellbeing and safety. This reflects the continued focus on doctor wellbeing following the COVID-19 pandemic.

In sum, the *Australasian Psychiatry* trainee article collection continues to expand with useful content that cements the journal’s role as a primary resource for those undertaking the RANZCP Fellowship programme.
